# Update analysis on the association between *Methionine synthase* rs1805087 A/G variant and risk of prostate cancer

**DOI:** 10.1038/s41598-020-70223-7

**Published:** 2020-08-07

**Authors:** Wei Zhang, Ze Zhang, Hao Wu, Kai Xu, Wei Yuan, Yuan-Yuan Mi, Li Shi, Li Zuo, Yun-Feng Shi

**Affiliations:** 1 Department of Oncology, Taizhou People’s Hospital, Taizhou, 225300 Jiangsu China; 2 Department of Urology, The Affiliated Changzhou No.2 People’s Hospital of Nanjing Medical University, 29 Xinglong Alley, Changzhou, 213003 Jiangsu Province China; 3 Department of Cardiology, Taizhou People’s Hospital, Taizhou, 225300 Jiangsu China; 4 Department of Urology, Affiliated Hospital of Jiangnan University, Wuxi, 214000 Jiangsu China; 5 Department of Urology, Wujin Hospital Affiliated Jiangsu University, Changzhou, China; 6 Department of Urology, Wujin Clinical College of Xuzhou Medical University, Changzhou, China

**Keywords:** Prostate cancer, Cancer epigenetics

## Abstract

Previous studies have investigated the association of the rs1805087 A/G variant of *Methionine synthase* gene with the susceptibility to prostate cancer (PCa). Nevertheless, the conclusions remain divergent. We performed a systemic analysis with odds ratios (ORs) and 95% confidence intervals (95% CIs) to assess *Methionine synthase* rs1805087 A/G variant and PCa risk. Furthermore, we utilized in silico analysis to investigate the relationship between *Methionine synthase* expression and the overall survival (OS) time. Totally, 10,666 PCa patients and 40,750 controls were included. We observed that *Methionine synthase* rs1805087 A/G variant is associated with an elevated risk of PCa (G-allele vs. A-allele: OR = 1.06, 95% CI = 1.01–1.11, *P* = 0.013; heterozygous model: OR = 1.08, 95% CI = 1.02–1.14, *P* = 0.009; dominant model: OR = 1.08, 95% CI = 1.02–1.14, *P* = 0.007). During stratified analysis, similar results were obtained in Asian populations, hospital-based, high quality studies and that with large sample size. Moreover, in silico analysis indicated the *Methionine synthase* expression is down-regulated in both young and old PCa subjects (*P* < 0.05). Compared with the normal subjects, the down-regulated expression of *Methionine synthase* was found in PCa cases with Gleason score 6 to 9. Our study showed that *Methionine synthase* rs1805087 A/G variant may be associated with susceptibility of PCa, especially in Asian populations, hospital-based studies and that with high quality and large sample size. Furthermore, *Methionine synthase* rs1805087 A/G variant may be related to the prognosis of PCa.

## Introduction

Prostate cancer (PCa) remains the most commonly occurring non-cutaneous carcinoma. It can be considered as the leading cause of cancer-related deaths among men in Western countries^1^. Although the incidence and mortality rate of PCa in Asian populations is lower than that in Western countries^2^, they have been increasing remarkably in recent years^3^. In China, the overall incidence is also increasing. PCa has become the most common solid tumor in urban male individuals. Therefore this highlights the need of strategies to prevent advanced PCa^4,5^. Up to now, various factors, such as age, hormone exposure and gene mutations, have been proved to be correlated with the development of PCa^6,7^. Particularly, growing evidence has indicated that genetic factors may play a crucial role in the etiology of PCa. For instance, the susceptibility of PCa would be increased around two to fivefold in patients suffering from Lynch syndrome. This disease is caused by gene mutations including *PMS2* (*Postmeiotic segregation increased—2*) and *MLH1* (*Mutl homolog—1*)^8–10^.

Previous studies have shown that folate metabolizing genes play a central role in carcinogenesis. They are involved in the process of DNA repair and methylation^11,12^. *Methionine synthase* is located on chromosome 1 (1q43) and with 34 exons. It encodes a core enzyme in folate pathway with 1,265 amino acids (molecular weight 140.5 kDa)^13^. *Methionine synthase* rs1805087 A/G variant is the most common mutation. It can lead to an aspartic acid to glycine transition at position 919 of the polypeptide chain^14–16^. Previous researches have indicated that this variant is involved in DNA methylation and elevation of homocysteine levels, therefore regulating the enzymatic activity of *Methionine synthase*^17^. The association of *Methionine synthase* rs1805087 A/G variant with susceptibility to PCa was investigated by several studies; however, their conclusions were divergent. In 2009, a systemic analysis evaluated the association between *Methionine synthase* rs1805087 A/G variant and susceptibility of PCa; nevertheless, they indicated no significant effects of this mutation on PCa risk^18^. From then on, another research assessed the association between this polymorphism and PCa susceptibility in Han Chinese population. They revealed that *Methionine synthase* rs1805087 A/G polymorphism was independently associated with PCa by down-regulating the potential of methylation^19^. The aim of the present study was to explore the association between *Methionine synthase* rs1805087 A/G variant and PCa susceptibility in larger sample size using multiple analyses to acquire convincing conclusion^18–32^.

## Methods

### Identification of relevant literature

We conducted a comprehensive literature search according to EMbase, PubMed, Google Scholar, Web of Science, and Chinese SinoMed databases. The keywords are as follows: (MTR OR METH OR methionine synthase) AND (variant OR single nucleotide polymorphism) AND (prostate cancer OR tumor) (last search updated on May 01, 2020). In addition, the reference lists of reviews or supplementary material of source documents were also retrieved for further research.

### Inclusion criteria and exclusion criteria

Studies were enrolled in our analysis according to the following criteria: (a) evaluating the relationship between PCa and *Methionine synthase* rs1805087 A/G polymorphism; (b) containing adequate information for all genotypes; and (c) case–control studies. Furthermore, studies should be removed if: (a) no control population was included; (b) research focuses on other diseases rather than cancer; (c) repeated previous publications.

### Data extraction and quality assessment

Relevant data were independently screened by two of the authors according to the selection criteria. The quality assessment of the included studies was investigated by Newcastle–Ottawa Scale (NOS). The NOS score ranges from 0 to 9 stars. A research can be considered as high-quality if it obtained seven or more stars. The following items were extracted: the first author’s name, publication year, race, source of control, score of NOS, genotype frequency, age range, Hardy–Weinberg Equilibrium (HWE) of cases and controls, sample size, and method. G-allele is a minor allele (mutated gene) for *Methionine synthase* rs1805087 A/G variant. On the other hand, the D-allele is a wild type and considered to be a low-risk allele. Five genetic models were selected in the current study: allelic comparison (G-allele *vs.* A-allele), homozygote model (GG *vs.* AA), heterozygote comparison (GA vs. AA), dominant contrast (GG + GA *vs.* AA), and recessive model (GG *vs.* GA + AA).

### Statistical analysis

The strength of relationship between *Methionine synthase* rs1805087 A/G variant and risk of PCa was assessed by odds ratios (ORs) and 95% confidence intervals (CIs). We adopt *Z*-test to measure statistical significance of ORs. Assumption of heterogeneity was calculated by Chi-square-based *Q*-test. Fixed-effect model (Mantel–Haenszel method) was used when *P* value of *Q*-test was more than 0.05; otherwise, the random-effect model was used^33,34^. Subgroup analyses were conducted by race, sample size of case, quality assessment, and source of controls. *P* value for HWE was evaluated by web-based program (https://ihg2.helmholtz-muenchen.de/cgibin/hw/hwa1.pl) ^35^. *P* value > 0.05 revealed an HWE balance. Sensitivity analysis was adopted to explore the effect of single study on the OR by sequential exclusion of individual study^36^. *I*^2^ value < 50% indicates no statistical heterogeneity among studies. We used the Begg's funnel plot and Egger's test to evaluate the publication bias^37,38^. *P* > 0.05 indicates no statistical significance. All the statistical process was conducted using software STATA v11.0 (Stata Corporation, College Station, TX).

### Study population and immunohistochemical staining (IHS)

Totally, 200 pathologically confirmed PCa patients were enrolled from our centers. Distribution of characteristics of these patients has been listed in our previous study^42^. The written informed consent was required from each participant. The above study protocol has been approved by ethics committee of the Affiliated Changzhou No.2 People's Hospital of Nanjing Medical University and Affiliated Hospital of Jiangnan University. IHS was used to test the *Methionine synthase* expression in PCa cases enrolled in our centers. We incubated the paraffin section of PRAD in hydrogen peroxide (1%), and then washed it in phosphate buffer saline (PBS). Goat serum was utilized to block binding of non-specific proteins. These sections were incubated with anti *Methionine synthase* antibody at 1:200. Immunoreactive site was brown using diaminobenzidine. All methods in the present study were conducted in accordance with the relevant guidelines and regulations.

### In silico analysis of *Methionine synthase* expression

We employed the online database to explore the expression of *Methionine synthase* in PCa and control counterparts (https://gemini.cancer-pku.cn/) ^39^. We also used The Cancer Genome Atlas (TCGA) samples to investigate the expression in PCa based on patients’ age, Gleason score, and nodal metastasis status. This database contains expression profiles of 52 PCa subjects and 496 controls (https://ualcan.path.uab.edu/analysis.html). We evaluated the effects of *Methionine synthase* rs1805087 A/G variant by SNAP tool (https://rostlab.org/services/snap/). String online server was also employed to assess the network of Methionine synthase interaction (https://string-db.org/) ^47^.

### Ethics approval and consent to participate

The present study was approved by ethics committee of the Affiliated Changzhou No.2 People's Hospital of Nanjing Medical University and ethics committee of Affiliated Hospital of Jiangnan University.

## Results

### Characteristics of relevant studies

**Figure 1:**
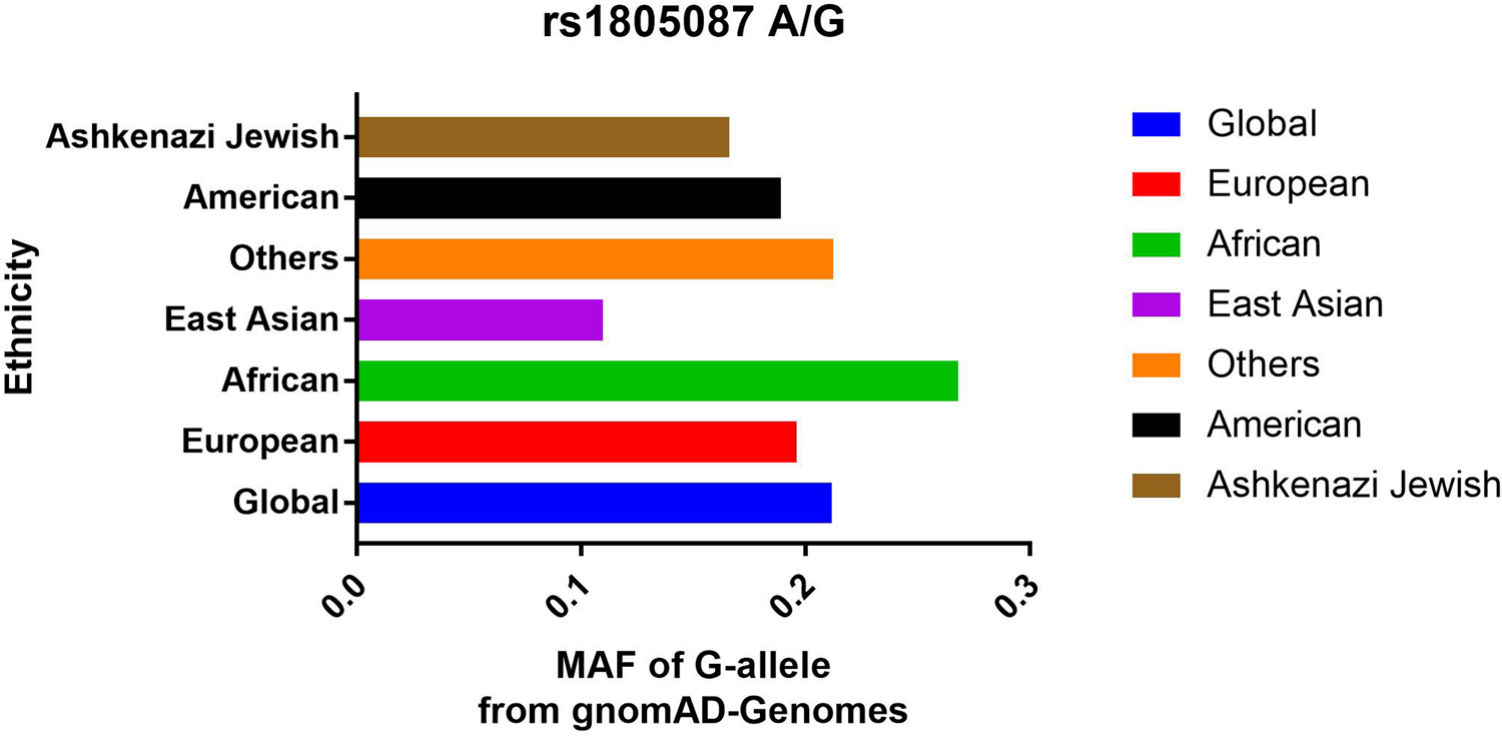
Minor allele frequency (MAF) of *Methionine synthase* rs1805087 A/G polymorphism in worldwide populations according to genome database.

A total of 15 case–control studies were included in our study. The PRIZMA statement has been presented in the Supplementary material Table 1. Totally, 10,666 PCa patients and 40,750 controls were included in the current analysis (Table 1). Subgroup analyses were based on the following criteria: (a) race: 10 studies were conducted in Europeans, 3 studies were based on Asian populations, only one study was on Africans and South Americans; (b) source of controls: there were 6 HB studies and 9 PB studies in the present analysis; (c) quality assessment: 12 studies were of high quality and 3 studies were of low quality. (d) Sample size of case: 9 were large sample size studies and the rest were small sample size studies. Furthermore, we investigated the minor allele frequencies (MAF) in worldwide populations according to genome database (https://www.ncbi.nlm.nih.gov/snp). In Global, G = 0.2118; Europeans, G = 0.1961; Americans, G = 0.1890; Africans, G = 0.2679; East Asians, G = 0.1098; Ashkenazi Jewish, G = 0.1660; and Others, G = 0.2123 (Fig. 1).

**Table 1:** Study characteristics of *Methionine synthase* rs1805087 A/G variant included in this analysis.

First author	Year	Race	Case	Control	Source	NOS	Age range (years)		Case				Control				Sample	Method
							Case	Control	GG	GA	AA	HWE	GG	GA	AA	HWE	size	
Ebrahimi	2017	Asian	100	100	HB	6	NA	NA	13	53	34	0.276	6	37	57	0.999	< 1,000	PCR–RFLP
Qu	2016	Asian	1817	2026	HB	8	66.7 ± 7.2	66.9 ± 6.8	20	316	1,481	0.496	15	319	1692	0.993	> 1,000	RT-PCR
López-Cortés	2013	SA	104	110	PB	8	NA	NA	3	9	92	< 0.001	1	4	105	0.001	< 1,000	PCR–RFLP
Jackson	2013	African	199	205	HB	7	67.8 ± 7.8	61.7 ± 10.7	20	82	97	0.664	24	82	99	0.274	< 1,000	Taqman
Weiner	2012	European	370	285	PB	6	69 ± 8	59 ± 17	15	134	221	0.339	16	96	173	0.580	< 1,000	RT-PCR
Cai	2010	Asian	217	220	HB	6	72.4 ± 12.2	72.8 ± 12.3	5	27	185	0.003	3	29	188	0.139	< 1,000	PCR–RFLP
Collin	2010	European	49	261	PB	8	62.6 ± 5.1	NA	1	16	32	0.534	10	77	174	0.686	< 1,000	GWAS
Murabito	2007	European	172	231	PB	7	66.0(43–85)	76.0(83–95)	7	55	110	0.970	9	69	153	0.728	< 1,000	GWAS
Stevens	2009	European	1,094	1,105	PB	7	50–69	30–108	42	351	701	0.814	53	324	728	0.032	> 1,000	Taqman
Yeager	2007	European	1,162	1,112	PB	7	NA	NA	48	376	738	0.990	38	340	734	0.858	> 1,000	GWAS
ProtecT	2008	European	1,600	2076	PB	7	NA	NA	52	515	1,033	0.207	84	637	1,355	0.402	> 1,000	Taqman
Eeles	2008	European	1,850	1886	PB	7	NA	NA	84	590	1,176	0.364	71	547	1,268	0.079	> 1,000	GWAS
Amundadottir	2006	European	1619	30,779	PB	7	mean 73.0	mean 52.0	60	466	1,093	0.242	1,044	9,160	20,575	0.532	> 1,000	GWAS
Marchal	2008	European	181	204	HB	7	70.7 ± 7.29	70.3 ± 7.82	9	54	118	0.391	11	55	138	0.088	< 1,000	Taqman
Kimura	2000	European	132	150	HB	9	65.6 ± 6.0	62.0 ± 11.4	4	41	87	0.753	4	44	102	0.773	< 1,000	PCR–RFLP

*GWAS* Genome-wide association study, *HWE* Hardy–Weinberg equilibrium of case and controls, *HB* Hospital-based, *NA* Not applicable, *PB* Population-based, *PCR–RFLP* polymerase chain reaction-restriction fragment length polymorphism, *RT-PCR* real-time PCR, *SA* South America.

### Systemic analysis

**Figure 2:**
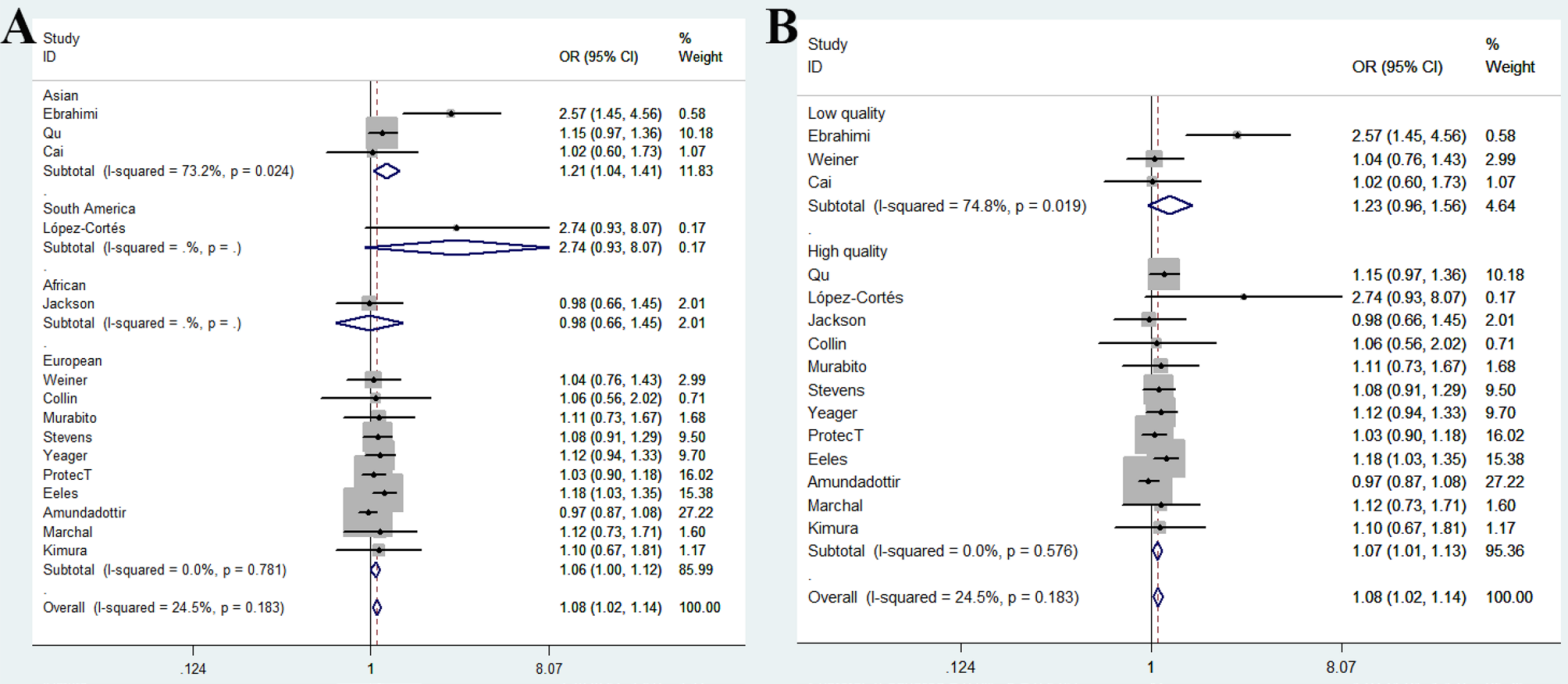
Forest plot shows odds ratio for the association between *Methionine synthase* rs1805087 A/G variant and PCa risk in subgroup analysis by race (**A**) and quality assessment (**B**) (GG + GA vs. AA, fixed-effects).

**Figure 3:**
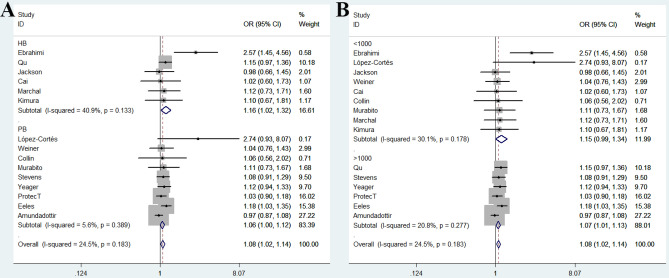
Forest plot of *Methionine synthase* rs1805087 A/G polymorphism in stratified analyses by source of control (**A**) and sample size (**B**).

In the overall analysis, we observed that *Methionine synthase* rs1805087 A/G variant was associated with increased risk of PCa (Table 2) under three genetic models. For GG + GA vs. AA comparison: OR = 1.08, 95% CI = 1.02–1.14, *P* value for heterogeneity = 0.183, *P* = 0.007, *I*^2^ = 24.5 (Fig. 2A); for G-allele vs. A-allele contrast: OR = 1.06, 95% CI = 1.01–1.11, *P*_heterogeneity_ = 0.122, *P* = 0.013, *I*^2^ = 30.9; and for GA vs. AA model: OR = 1.08, 95% CI = 1.02–1.14, *P* value for heterogeneity = 0.353, *P* = 0.009, *I*^2^ = 8.9. In stratification analysis by race, similar positive results were revealed in Asian descendants. During GG vs. AA model: OR = 1.93, 95% CI = 1.14–3.26, *P*_heterogeneity_ = 0.390, *P* = 0.014, *I*^2^ = 0; for GG vs. GA + AA model: OR = 1.72, 95% CI = 1.02–2.89, *P*_heterogeneity_ = 0.767, *P* = 0.041, *I*^2^ = 0. In stratified analysis based on quality assessment, a positive relationship was revealed between *Methionine synthase* rs1805087 A/G variant and PCa risk in high quality studies (allelic contrast: OR = 1.05, 95% CI = 1.01–1.11, *P*_heterogeneity_ = 0.417, *P* = 0.029, *I*^2^ = 2.8; heterozygous model: OR = 1.07, 95% CI = 1.01–1.13, *P*_heterogeneity_ = 0.696, *P* = 0.020, *I*^2^ = 0; dominant comparison: OR = 1.07, 95% CI = 1.01–1.13, *P*_heterogeneity_ = 0.576, *P* = 0.018, *I*^2^ = 0), but not in low quality studies (allelic contrast: OR = 1.27, 95% CI = 0.81–1.98, *P*_heterogeneity_ = 0.019, *P* = 0.295, *I*^2^ = 74.8; heterozygous model: OR = 1.31, 95% CI = 0.80–2.16, *P*_heterogeneity_ = 0.046, *P* = 0.281, *I*^2^ = 67.5; dominant comparison: OR = 1.35, 95% CI = 0.79–2.32, *P*_heterogeneity_ = 0.019, *P* = 0.270, *I*^2^ = 74.8) (Fig. 2B). Furthermore, in subgroup analysis based on source of control and sample size, similar findings were obtained between *Methionine synthase* rs1805087 A/G variant and PCa susceptibility in hospital-based studies (allelic contrast: OR = 1.15, 95% CI = 1.02–1.29, *P*_heterogeneity_ = 0.137, *P* = 0.018, *I*^2^ = 40.3; heterozygous model: OR = 1.15, 95% CI = 1.01–1.32, *P*_heterogeneity_ = 0.245, *P* = 0.041, *I*^2^ = 25.3; dominant comparison: OR = 1.16, 95% CI = 1.02–1.32, *P*_heterogeneity_ = 0.133, *P* = 0.023, *I*^2^ = 40.9, Fig. 3A) and studies with larger sample size (allelic contrast: OR = 1.05, 95% CI = 1.00–1.11, *P*_heterogeneity_ = 0.226, *P* = 0.039, *I*^2^ = 27.8; GA vs. AA model: OR = 1.07, 95% CI = 1.00–1.13, *P* value for heterogeneity = 0.309, *P* = 0.036, *I*^2^ = 16.2; GG + GA vs. AA model: OR = 1.07, 95% CI = 1.01–1.13, *P* value for heterogeneity = 0.277, *P* = 0.029, *I*^2^ = 20.8, Fig. 3B).

**Table 2:** Stratified Stratification analysis of *Methionine synthase* rs1805087 A/G polymorphism on the susceptibility to PCa.

Variables	No	Cases/controls	G-allele vs. A-allele							GA vs. AA				GG vs. AA				GG + GA vs. AA				GG vs. GA + AA			
			OR (95% CI)		*P*		*P*_heter_		*I*^2^	OR (95% CI)	*P*	*P*_heter_	*I*^2^	OR (95% CI)	*P*	*P*_heter_	*I*^2^	OR (95% CI)	*P*	*P*_heter_	*I*^2^	OR (95% CI)	*P*	*P*_heter_	*I*^2^
Total	15	10,666/40,750	1.06 (1.01–1.11)		0.013		0.122		30.9	1.08 (1.02–1.14)	0.009	0.353	8.9	1.07 (0.93–1.22)	0.346	0.376	6.8	1.08 (1.02–1.14)	0.007	0.183	24.5	1.04 (0.91–1.19)	0.589	0.573	0
Race																									
European	10	8,229/38,089	1.04 (0.99–1.10)		0.106		0.758		0	1.06 (1.00–1.13)	0.052	0.771	0	1.02 (0.89–1.18)	0.749	0.673	0	1.06 (1.00–1.12)	0.060	0.781	0	1.00 (0.87–1.16)	0.659	0.974	0
Asian	3	2,134/2,346	1.33 (0.94–1.88)		0.109		0.050		66.6	1.31 (0.84–2.05)	0.233	0.044	68.0	1.93 (1.14–3.26)	0.014	0.390	0	1.38 (0.87–2.20)	0.174	0.024	73.2	1.72 (1.02–2.89)	0.041	0.767	0
South America	1	104/110	2.77 (1.05–7.29)		0.039		–		–	2.57 (0.77–8.62)	0.127	–	–	3.42 (0.35–33.49)	0.290	–	–	2.74 (0.93–8.07)	0.067	–	–	3.24 (0.33–31.63)	0.312	–	–
African	1	199/205	0.95 (0.71–1.28)		0.746		–		–	1.02 (0.67–1.55)	0.923	–	–	0.85 (0.44–1.64)	0.629	–	–	0.98 (0.66–1.45)	0.928	–	–	0.84 (0.45–1.58)	0.593	–	–
Source																									
PB	9	8,020/37,845	1.04 (0.99–1.10)		0.095		0.287		17.5	1.06 (1.00–1.13)	0.053	0.474	0	1.03 (0.89–1.19)	0.688	0.467	0	1.06 (1.00–1.12)	0.057	0.389	5.6	1.01 (0.87–1.17)	0.901	0.461	0
HB	6	2,646/2,905	1.15 (1.02–1.29)	0.018		0.137		40.3		1.15 (1.01–1.32)	0.041	0.245	25.3	1.31 (0.92–1.87)	0.138	0.303	17.2	1.16 (1.02–1.32)	0.023	0.133	40.9	1.22 (0.86–1.73)	0.273	0.568	0
Sample size																									
Large	6	9,142/38,984	1.05 (1.00–1.11)	0.039		0.226		27.8		1.07 (1.00–1.13)	0.036	0.309	16.2	1.06 (0.92–1.23)	0.424	0.258	23.4	1.07 (1.01–1.13)	0.029	0.277	20.8	1.04 (0.90–1.21)	0.588	0.258	23.4
Small	9	1524/1766	1.11 (0.97–1.26)	0.115		0.121		38.1		1.16 (0.99–1.37)	0.064	0.390	5.4	1.09 (0.78–1.52)	0.604	0.386	5.9	1.15 (0.99–1.34)	0.069	0.178	30.1	1.02 (0.74–1.41)	0.909	0.662	0
Quality																									
High	12	9,979/40,145	1.05 (1.01–1.11)	0.029		0.417		2.8		1.07 (1.01–1.13)	0.020	0.696	0	1.05 (0.91–1.21)	0.479	0674	0	1.07 (1.01–1.13)	0.018	0.576	0	1.03 (0.90–1.18)	0.678	0.676	0
Low	3	687/605	1.27 (0.81–1.98)	0.295		0.019		74.8		1.31 (0.80–2.16)	0.281	0.046	67.5	1.56 (0.54–4.49)	0.411	0.047	67.3	1.35 (0.79–2.32)	0.270	0.019	74.8	1.16 (0.68–1.97)	0.588	0.142	48.7

*No.* Number of case–control studies, *P*_*heter*_*P* value for heterogeneity. *HB* Hospital-based, *PB* population-based.

### In silico analysis and IHS for *Methionine synthase* expression

**Figure 4:**
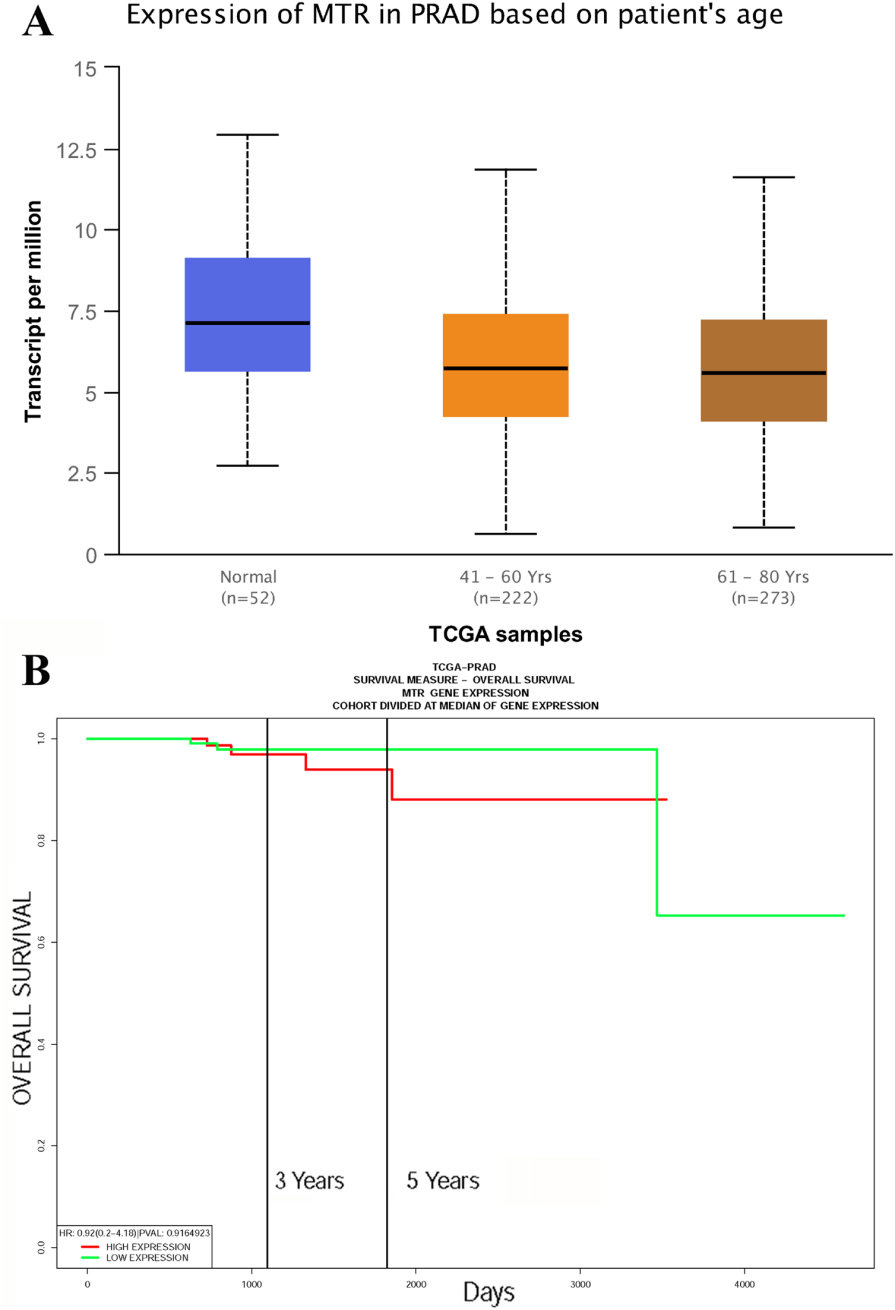
In silico analysis of the expression of *Methionine synthase*. The expression of *Methionine synthase* is down-regulated in prostate adenocarcinoma tissue (both =  < 60 years and > 60 years group, **A**). The relationship between the expression of *Methionine synthase* and OS time among PCa patients (**B**).

**Figure 5:**
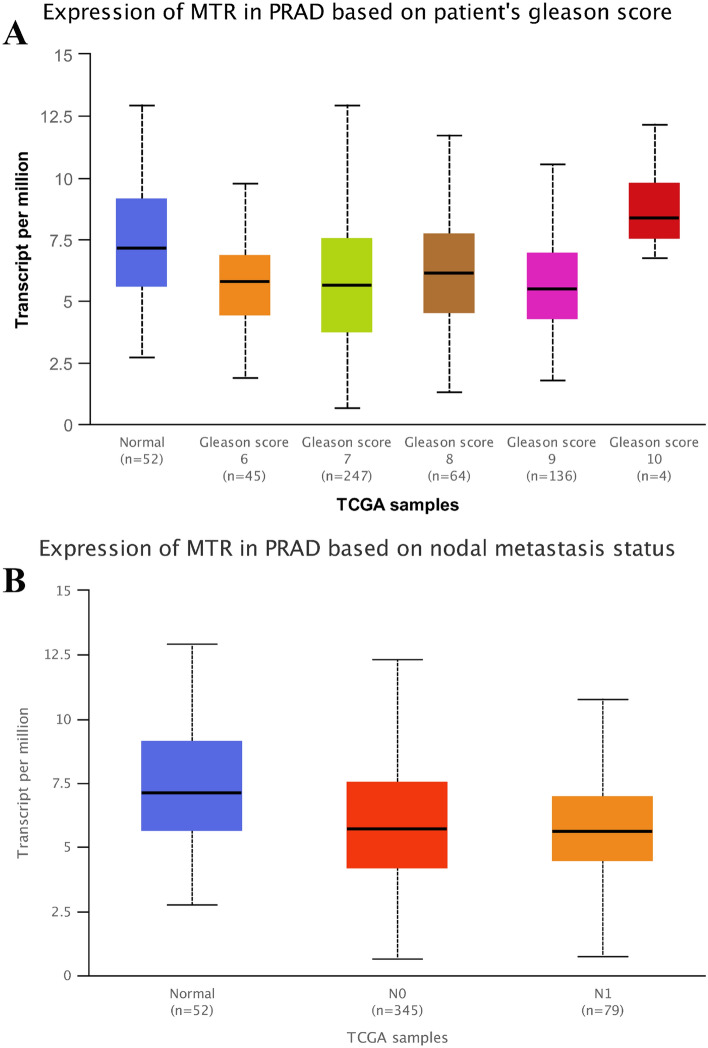
The expression of *Methionine synthase* in PCa based on patients' Gleason score (**A**) and nodal metastasis status (**B**).

**Figure 6:**
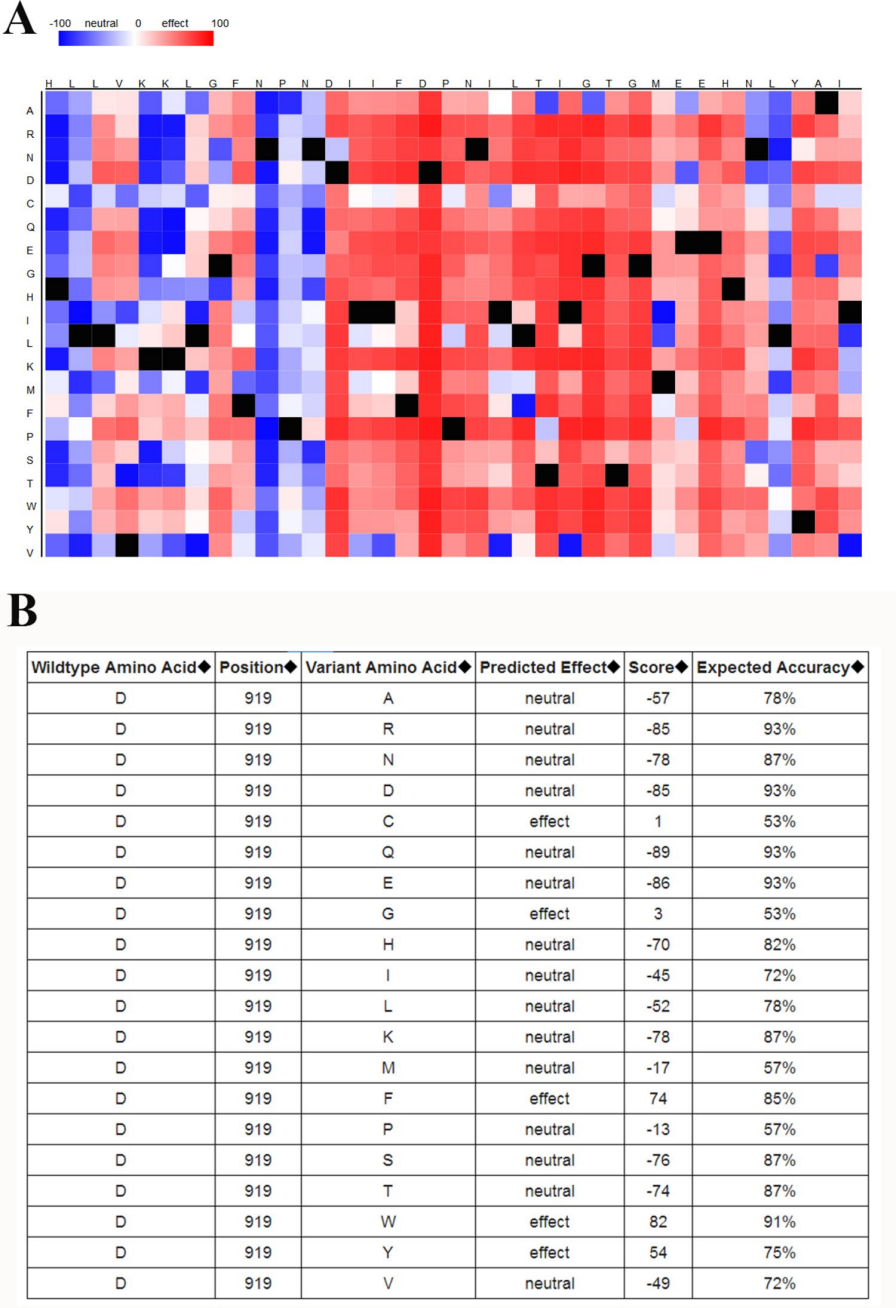
Evaluation of *Methionine synthase* rs1805087 A/G variant by SNAP tool (**A**). The mutation score is 3, which indicate that *Methionine synthase* rs1805087 A/G variant is deleterious and effect (**B**).

**Figure 7:**
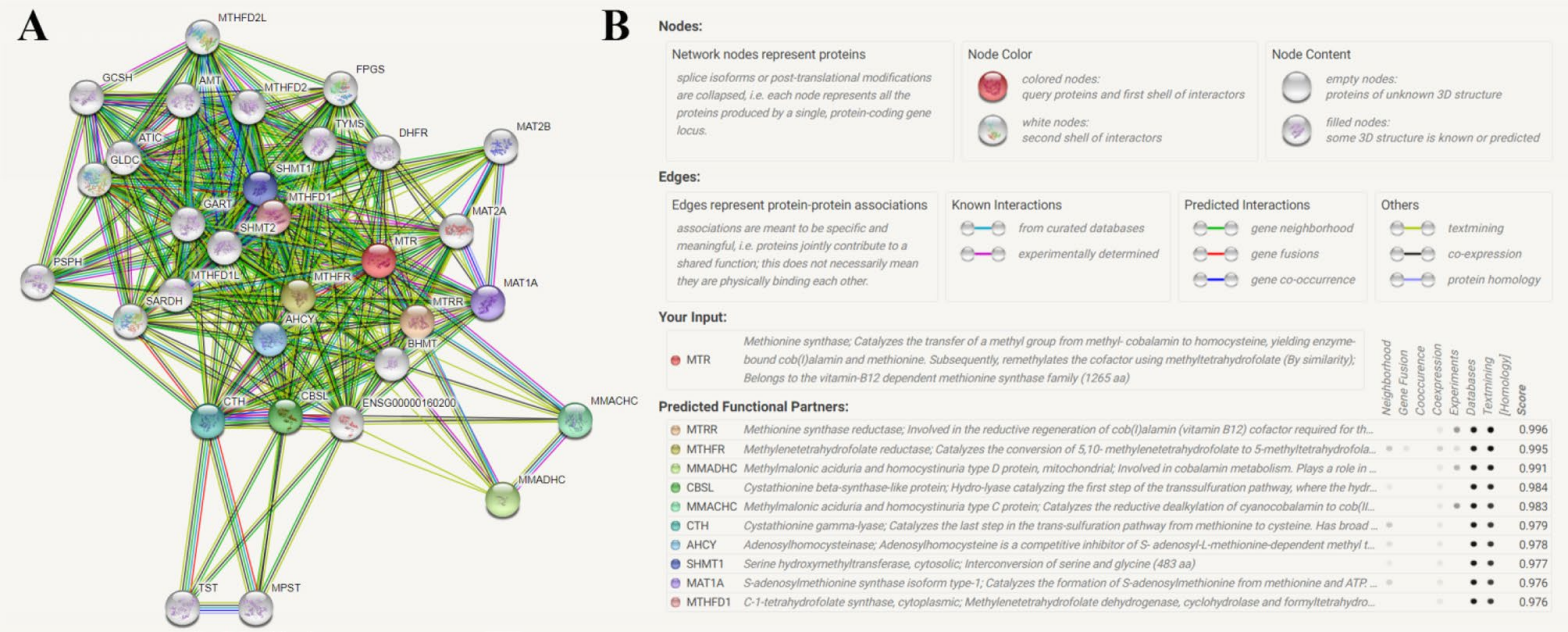
Methionine synthase crosstalk with other protein evaluated by String server. At least 30 proteins are predicted to participate in the protein–protein interaction with Methionine synthase in *Homo sapiens* (**A**). The top 10 proteins are: *MTRR* Methionine synthase reductase, *MTHFR* Methylenetetrahydrofolate reductase, *MMADHC* Methylmalonic aciduria and homocystinuria type D protein, mitochondrial, *CBSL* Cystathionine beta-synthase-like protein, *MMACHC* Methylmalonic aciduria and homocystinuria type C protein, *CTH* Cystathionine gamma-lyase, *AHCY* Adenosylhomocysteinase, *SHMT1* Serine hydroxymethyltransferase, *MAT1A* S-adenosylmethionine synthase isoform type-1, *MTHFD1* Methylenetetrahydrofolate dehydrogenase (**B**).

**Figure 8:**
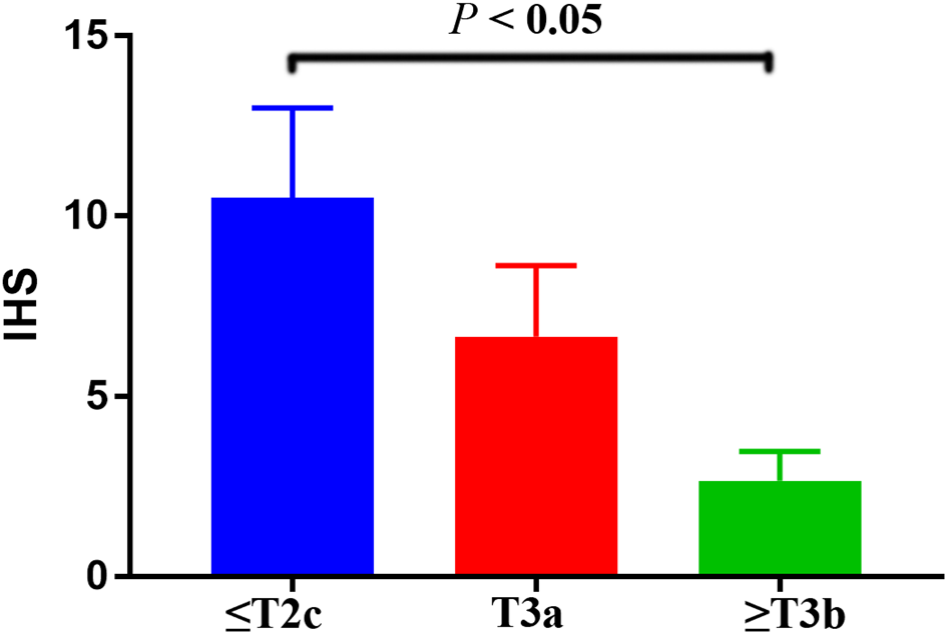
Tissue expression of *Methionine synthase* in PCa subjects. Compared with the earlier stage, the down-regulated expression of *Methionine synthase* is found in more advanced PCa participants (stage >  = T3b versus =  < T2c, *P* < 0.05).

We employed in silico tool to investigate the expression of *Methionine synthase* among 496 PCa patients and 52 controls. The overall result showed that the methionine synthase expression is down-regulated in both young and old PCa subjects (*P* < 0.05, Fig. 4A). In addition, we assessed whether the level of *Methionine synthase* expression affected the OS time of patients with PCa. As shown in Fig. 4B, PCa patients with high expression of *Methionine synthase* may have a shorter OS time in the first 5 years. Nevertheless, with the passage of time, this positive relationship would not be statistically significant (*P* > 0.05). TGCA database^40^ showed evidence that the expression of *Methionine synthase* in PCa cases with Gleason score 6 to 9 was lower than that in normal subjects (*P* < 0.05, Fig. 5A). In addition, the *Methionine synthase* expression was both down-regulated in PCa patients with and without lymph node metastasis (*P* < 0.05, Fig. 5B). In order to assess whether the *Methionine synthase* rs1805087 A/G variant could impact the protein expression, we used SNAP tool to predict the mutation of *Methionine synthase* (Fig. 6A). The mutation score is 3, which indicate that *Methionine synthase* rs1805087 A/G variant is deleterious and effect (Fig. 6B). As described in Fig. 7A, at least 30 proteins are predicted to participate in the protein–protein interaction with Methionine synthase in *Homo sapiens*. The top 10 proteins are: MTRR: Methionine synthase reductase; MTHFR: Methylenetetrahydrofolate reductase; MMADHC: Methylmalonic aciduria and homocystinuria type D protein, mitochondrial; CBSL: Cystathionine beta-synthase-like protein; MMACHC: Methylmalonic aciduria and homocystinuria type C protein; CTH: Cystathionine gamma-lyase; AHCY: Adenosylhomocysteinase; SHMT1: Serine hydroxymethyltransferase; MAT1A: S-adenosylmethionine synthase isoform type-1; MTHFD1: Methylenetetrahydrofolate dehydrogenase (Fig. 7B). In order to further investigate the *Methionine synthase* expression in PCa tissues, we adopted IHS to assess its expression among PCa participants in our hospitals. Compared with the earlier stage, the down-regulated expression of *Methionine synthase* was found in more advanced PCa participants (stage >  = T3b versus =  < T2c, *P* < 0.05, Fig. 8).

### Sensitivity analysis and publication bias

**Figure 9:**
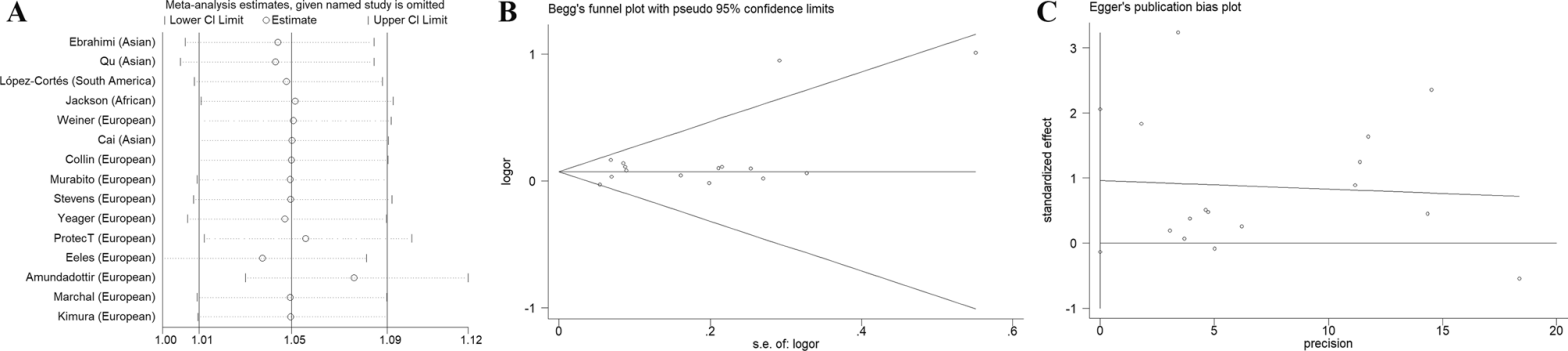
Publication bias analysis of *Methionine synthase* rs1805087 A/G variant and PCa risk. Sensitivity results were assessed by removing every single study in turn (**A**). No evidence of publication bias can be revealed through both Begg's funnel plot (**B**) and Egger's test(**C**).

The sensitivity analysis was conducted to investigate the effect of individual study on the OR. As described in Fig. 9A, no single study would significantly impact the overall OR. Both the Begg’s funnel plot (Fig. 9B) and Egger’s tests (Fig. 9C) were performed to assess the publication bias. No evidence of publication bias was revealed among any of the genetic models. For G-allele vs. A-allele: t = 2.01, *P* = 0.066; GA vs. AA: t = 2.03, *P* = 0.064; GG vs. AA: t = 1.95, *P* = 0.073; GG + GA vs. AA: t = 2.06, *P* = 0.060; and GG vs. GA + AA model: t = 1.92, *P* = 0.077.

## Discussion

Identification of genetic mutations that related to susceptibility of carcinoma is useful to predict cancer risk and reveal the pathogenesis of various tumors^41,42^. PCa remains one of the most commonly occurring carcinomas among men in Western countries. It has been clarified that several risk factors, such as family history, hormone exposure, lifestyle, may be associated with susceptibility of PCa^43–46^. Previous articles showed that *Methionine synthase* may be involved in the development of PCa^29^. However, the assoiation between *Methionine synthase* rs1805087 A/G variant and PCa risk remains ambiguous. For instance, a case–control study based on Iran descendants found that *Methionine synthase* rs1805087 A/G variant could influence stability and activity of its expression^22^. This polymorphism might be related to PCa risk in male individuals. However; another researchers did not indicate similar conclusions^26^. A meta-analysis conducted 10 years ago revealed no significant associations between *Methionine synthase* rs1805087 A/G variant and PCa risk^18^. From then on, more and more researchers ascertained their results in different populations^19,20,22,23,25,26^. Hence, in our systemic analysis, all eligible studies based on the inclusion criteria were summerized to ascertain a precise conclusion on the association between *Methionine synthase* rs1805087 A/G variant and PCa risk. Totally, 10,666 PCa patients and 40,750 controls were included to investigate the *Methionine synthase* polymorphism.

In our analysis, 15 case–control studies were included. The overall results revealed a positive association between *Methionine synthase* rs1805087 A/G variant and susceptibility of PCa under three genetic models. The conclusions acquired from our study were: person carrying the *Methionine synthase* G-allele may have an increased PCa risk. In stratification analysis by race, we observed similar positive results in Asian populations. *Methionine synthase* rs1805087 A/G variant might elevate PCa susceptibility as seen in high quality studies, hospital-based studies, and that with large sample size. Furthermore, we used online gene expression mini-database to investigate *Methionine synthase* expression in PCa and control counterparts. We found that *Methionine synthase* expression is down-regulated in both young and old PCa subjects. Compared with the normal subjects, the down-regulated expression of *Methionine synthase* was found in PCa cases with Gleason score 6 to 9, which was consistent with results of our IHS analysis. We also evaluated whether the *Methionine synthase* expression level influence the PCa patients' OS time. PCa patients with high *Methionine synthase* expression may have a shorter OS time in the first 5 years. However, with the passage of time, this positive relationship would not be statistically significant. In addition, we used SNAP tool to evaluate the variation of *Methionine synthase.* The mutation score is 3, which indicate that *Methionine synthase* rs1805087 A/G variant is deleterious and effect.

Although considerable resources have been generated to evaluate association between *Methionine synthase* rs1805087 A/G variant and PCa susceptibility, there are some limitations that should be considered. To start with, only three Asian studies involving *Methionine synthase* rs1805087 A/G variant and PCa risk were retrieved, which indicates that the total participants count for Asian population remains relatively low for more comprehensive analysis. Second, all included case–control studies were retrospective, which may cause selection bias during the process of analysis. Third, lacking of some original data such as family history, lifestyle, and smoking exposure, may limit the efficacy to further calculate adjusted OR. Fourth, studies with only English or Chinese language were included; therefore, some articles written in other languages may be missing, which may cause some bias in risk estimation. In spite of the limitations, there are several advantages in our analysis. First, HWE is very significant while evaluating genetic variations. *P* value for HWE is more than 0.05 in most of the included studies, which shows that the conclusion from the included studies is very stable. Second, studies with larger sample size particularly improved the statistical efficiency. Third, we indicated no publication bias while evaluating *Methionine synthase* rs1805087 A/G variant, so the conclusions of the present analysis are more convincing. As described in Fig. 7A, at least 30 proteins are predicted to participate in the protein–protein interaction with *Methionine synthase* in *Homo sapiens*. Nevertheless, there is not enough research on their further mechanism in PCa. Further researches are warranted to explore the interactions in more details.

## Conclusions

Taken together, our study indicated that *Methionine synthase* rs1805087 A/G variant is associated with PCa susceptibility, especially in Asian descendants, hospital-based studies, high quality studies and that with large sample size. Moreover, *Methionine synthase* rs1805087 A/G variant may be related to the prognosis of PCa. Further studies containing more information such as lifestyle and smoking exposure, are warranted to assess this association in details.

## Availability of data and materials

All the data analyzed in the present study is included in the manuscript.

## Supplementary information

Supplementary file1.

## Abbreviations

CIs: Confidence intervals
GWAS: Genome-wide association study
HWE: Hardy–Weinberg equilibrium of controls
IHS: Immunohistochemical staining
MAF: Minor allele frequencies
HB: Hospital-based
MLH1: Mutl homolog—1
NA: Not applicable
OS: Overall survival
PB: Population-based
NOS: Newcastle–Ottawa Scale
ORs: Odds ratios
PBS: Phosphate buffer saline
PCR–RFLP: Polymerase chain reaction-restriction fragment length polymorphism
PCa: Prostate cancer
PMS2: Postmeiotic segregation increased—2)
RT-PCR: Real-time PCR
TCGA: The cancer genome Atlas
vs: Versus
